# MINISTOP 2.0: a smartphone app integrated in primary child health care to promote healthy diet and physical activity behaviours and prevent obesity in preschool-aged children: protocol for a hybrid design effectiveness-implementation study

**DOI:** 10.1186/s12889-020-09808-w

**Published:** 2020-11-23

**Authors:** Hanna Henriksson, Christina Alexandrou, Pontus Henriksson, Maria Henström, Marcus Bendtsen, Kristin Thomas, Ulrika Müssener, Per Nilsen, Marie Löf

**Affiliations:** 1 Department of Health, Medicine and Caring Sciences, Linköping University, 581 83 Linköping, Sweden; 2 Department of Biosciences and Nutrition, Karolinska Institutet, 141 83 Huddinge, Stockholm, Sweden

**Keywords:** mHealth, Diet, Physical activity, Childhood obesity, Preschool, Randomised controlled trial, Primary child health care

## Abstract

**Background:**

Childhood obesity is still a major health problem in many countries, including Sweden. Childhood obesity and obesity-related behaviours in childhood, such as low physical activity and unhealthy eating habits, tend to track into adulthood, which highlights the need for early prevention. Our aims are to evaluate whether a parent-oriented mobile health app (the MINISTOP 2.0 app) integrated into primary child health care can improve diet and physical activity behaviours and reduce the prevalence of overweight and obesity in preschool-aged children as well as to evaluate the implementation among child health care nurses and parents.

**Methods:**

This trial uses a hybrid type 1 effectiveness-implementation design. Families (*n* = 500) who attend a routine visit to one of 15–20 primary child health care centres throughout Sweden, when their child is 2.5 years, are offered participation in a randomised controlled trial (effectiveness evaluation). After acceptance, families will be randomised (1:1) to control or intervention groups. The intervention group receives a 6-month parent-oriented smartphone intervention aimed at improving the dietary and activity behaviours of their child (the MINISTOP 2.0 app) and the control group receives routine child health care. Dietary habits, physical activity and screen time (primary outcomes), body weight and height in children, and parental self-efficacy (secondary outcomes) are measured at baseline and at 6 months post randomisation. Implementation outcomes (i.e. perceived acceptability, appropriateness, and feasibility) of the intervention will be assessed among primary child health care nurses and parents in the trial through questionnaires and qualitative interviews.

**Discussion:**

This trial will evaluate whether the MINISTOP 2.0 app can be used in primary child health care to improve diet and physical activity behaviours, and prevent overweight and obesity, in preschool-aged children. If effectiveness is proven, and the MINISTOP 2.0 app is considered acceptable, appropriate and feasible, it can be implemented nationally as part of the preventive strategies to combat childhood obesity provided by routine child health care.

**Trial registration:**

The trial was registered at the Clinicaltrials.gov register platform (ID NCT04147039) on 31 October 2019.

**Supplementary Information:**

The online version contains supplementary material available at 10.1186/s12889-020-09808-w.

## Background

Childhood obesity is still a major global health issue [1]. In 2019, 38 million children under the age of five were overweight or obese [2]. Sweden is no exception with 10–15% of 4-year-olds being overweight or obese [3, 4]. Although increases in the prevalence of obesity have generally been shown to level off in developed countries [1, 5], recent data indicate that obesity is still increasing among school-aged children in Sweden [6]. As in other high-income countries, there is a socioeconomic gradient of obesity in Sweden, with a higher prevalence already in young childhood among socioeconomically disadvantaged or migrant families [7–9]. In addition, only 33 and 20% of Swedish preschool-aged children fulfil recommendations for physical activity and the intake of fruit and vegetables, respectively [10, 11]. Childhood obesity and obesity-related behaviours, such as low physical activity and unhealthy diet, tend to track into adulthood [12–14] where they are associated with increased risk of disease, impaired psychosocial well-being and premature death [5, 15–17]. Given these serious health consequences, prevention of obesity and promotion of health behaviours in childhood are major public health priorities.

Preschool age (2–5 years) has been identified as a critical period to intervene [18], and primary child health care has been regarded as an important setting for obesity treatment and prevention efforts [19]. However, to date, interventions for this age group have only reported limited effectiveness for obesity prevention and improvement of health behaviours [19–24]. Furthermore, effective interventions that can decrease childhood obesity inequalities in preschool-aged children are lacking [9]. Collectively, these findings highlight the need for innovative solutions to facilitate obesity prevention in young children.

During the last decade, digitalisation and advancements in mobile phone technology have radically changed communication and offer new opportunities to improve how health care services are delivered. For instance, mobile phone technologies have been successfully integrated into interventions to promote healthy diets, physical activity and weight loss [25, 26]. The benefits of using mobile phone based (mHealth) versus more traditional face-to-face interventions are that they can be delivered at any time or place and participants do not have to attend a clinic (or the number of visits can be reduced). Also, given the ubiquitous use of mobile phones, irrespectively of socioeconomic status, mHealth offers the ability to deliver relatively low-cost interventions at scale [27].

A novel mHealth obesity prevention trial in preschool-aged children, the MINISTOP (Mobile-based Intervention Intended to Stop Obesity in Preschoolers) 1.0 trial, was conducted 2013–2015 in a population-based sample of Swedish children [28]. The MINISTOP 1.0 smartphone app had a high participation rate and usage by parents. The efficacy evaluation showed no group difference in fat mass index; however, children in the app group had higher odds of improving a composite score of six dietary and activity behaviours (OR: 2.0; 95% CI 1.2–3.1; *p* = 0.008). The effect of the intervention on dietary and activity behaviours was more pronounced in the children with a higher fat mass index (*p* = 0.019), which is promising as these children need it the most. It is noteworthy that the effect size was greater or equal to more labour-intense traditional, face-to-face delivered interventions [23, 24]. However, the MINISTOP 1.0 app was provided to the families by researchers, which is not feasible in real life conditions. Therefore, the next step is to evaluate the MINISTOP app in a primary child health care context in order to investigate its effectiveness and implementation. To optimise accessibility, the MINISTOP 1.0 app has been translated and tailored for priority populations, such as children with a migrant background (≈25% of all Swedish children) [29] who are less likely to participate in population-based health strategies [30]. Furthermore, the MINISTOP 1.0 app has been modified based on feedback and requirements from users, i.e. parents and health care providers. The effectiveness and implementation of this revised version of the app (called MINISTOP 2.0) will be evaluated in real life conditions, i.e. in routine child health care. This project (called the MINISTOP 2.0 trial) has been initiated in close collaboration with Swedish primary child health care. The MINISTOP 2.0 trial is one of seven mHealth interventions in the MoBILE research programme (principal investigator: ML) aiming to promote healthy lifestyle habits [31].

### Aims

This protocol describes the study design and methodology of the MINISTOP 2.0 trial, using a hybrid type 1 effectiveness-implementation study design. The overall aim of the MINISTOP 2.0 trial is to evaluate whether an mHealth intervention, embedded in the routine services of primary child health care in Sweden, can be used to promote healthy dietary and activity behaviours and to decrease the prevalence of overweight and obesity in preschool-aged children. Specifically, we aim to evaluate:

1. The effectiveness of the MINISTOP 2.0 app in terms of effects on: a) key dietary indicators, assessed as intake of fruit and vegetables, sweet and savoury treats and sweet drinks (primary outcome); b) physical activity, assessed as time spent on moderate-to-vigorous physical activity (primary outcome); c) screen time (primary outcome); d) body mass index (BMI) (secondary outcome); and e) parental self-efficacy (secondary outcome).
2. The implementation of the MINISTOP 2.0 app with regard to perceived acceptability, appropriateness, and feasibility among parents and primary health care nurses.

## Methods

### Setting and study design

The trial is based on a hybrid type 1 study design which combines an effectiveness study with an implementation study [32]. Thus, a two-arm parallel groups randomised controlled trial will investigate the effectiveness of the MINISTOP 2.0 app on health behaviours, BMI, and parental self-efficacy. In addition, implementation will be evaluated in terms of perceived acceptability, appropriateness, and feasibility among parents and nurses through questionnaires and interviews. This type of study design involves the testing of the effectiveness of an intervention while simultaneously obtaining information on its delivery and its potential for implementation under real-world circumstances [32]. This protocol is in accordance with the SPIRIT 2013 statement [33] and the intervention will be described according to the CONSORT – EHEALTH checklist (v1.6.1) [34]. Important protocol modifications will be reported in the trial registration as well as in the publications reporting the study outcomes.

### The intervention – the MINISTOP 2.0 app

**Fig. 1:**
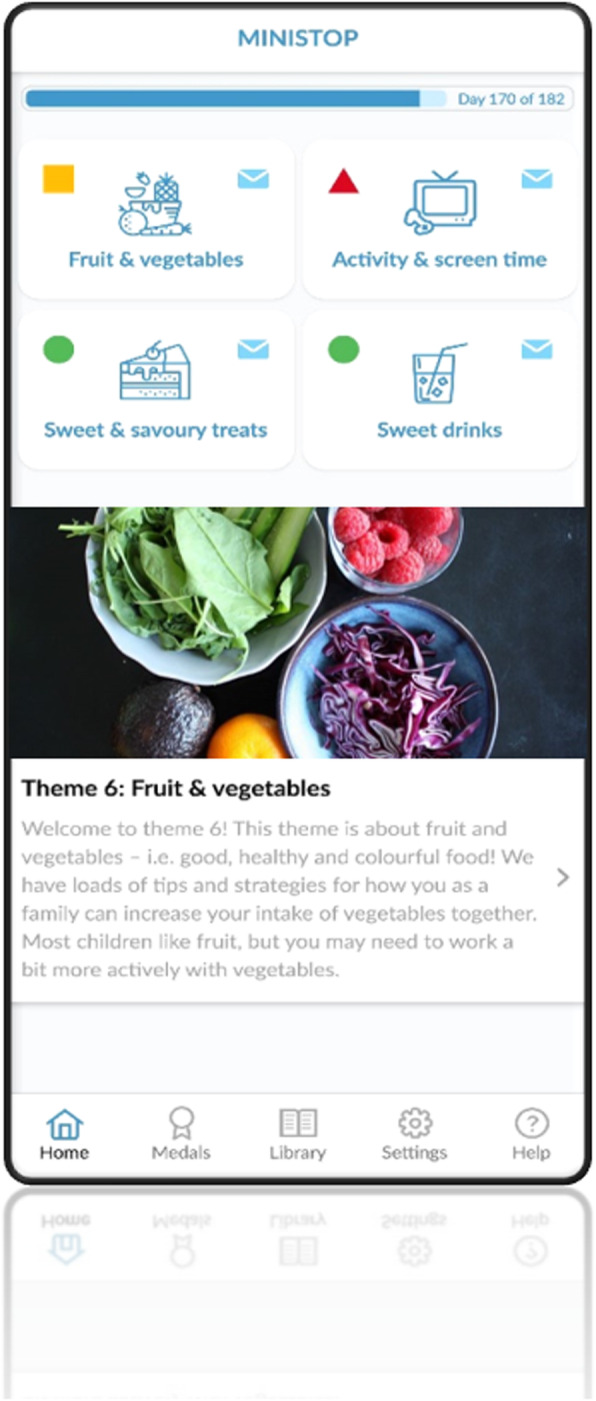
Screenshot of the MINISTOP 2.0 app

The MINISTOP 2.0 app is a parent-oriented comprehensive 6-month smartphone intervention aimed at improving dietary- and physical activity behaviours and preventing the prevalence of overweight and obesity in preschool-aged children. A screenshot of the MINISTOP 2.0 app is shown in Fig. 1. The app is grounded in social cognitive theory and key behavioural change techniques [35, 36] such as shaping knowledge (e.g. information about healthy eating and physical activity), goal setting, identification of barriers, and self-monitoring and feedback.

#### Development of the intervention

The MINISTOP 2.0 app is built on the MINISTOP 1.0 technical platform and content, and is both Android and IOS compatible [28]. New features have been added and translation of content and other modifications required have been performed to optimise participation. Two pilot studies were conducted to test the content, confirm selected modules, add additional themes, and to obtain other relevant information that could be integrated into the MINISTOP 2.0 app. In a first pilot study, individual interviews with nurses (*n* = 15) who worked in primary child health care were conducted as well as focus groups with parents (one group was Swedish speaking, one Somali and one Arabic), also recruited from a primary child health care setting. The focus groups with migrant parents were conducted with a certified interpreter. In a second pilot study, ten parents and six nurses were asked to test the intervention during a two-week period and provide comments on the features and content of the app using a questionnaire. Information retrieved from the pilot studies was used to modify the app to optimise content and participation. Details and results of these two pilot studies will be reported elsewhere.

#### Content and use of the intervention

The MINISTOP 2.0 app consists of a comprehensive program with information grounded in evidence-based recommendations for preschool-aged children concerning healthy eating [37], physical activity [38, 39] and screen time [38, 39]. The app consists of four modules: general information; push notifications; self-monitoring and feedback; and a library with general facts, recipes and tips on physical activity. The content is built around a series of themes which change every other week and addresses 13 relevant topics: 1) everyday food, 2) breakfast, 3) healthy snacks, 4) physical activity and screen time, 5) sweets and snacks, 6) fruit and vegetables, 7) beverages, 8) snacking, 9) fast food, 10) sleep, 11) meals outside the home, 12) food as a reward / on special occasions, and 13) dental health. Each theme is structured into three parts (basic facts, practical tips, strategies), and while a new theme is introduced every second week, all previous themes are still stored and fully accessible in the library. In the Arabic and Somali versions of the app, the themes are also available as audio files.

Participants receive one push notification every day, related to the current theme, or when a new theme is initiated. The push notifications consist of short messages with support and tips on healthy habits (diet, physical activity, screen time and dental health), tips and strategies for behavioural change, encouragement, key messages or reminders.

In the self-monitoring and feedback module of the app, parents can monitor their child’s physical activity and screen time, and their intake of fruit and vegetables, sweets and snacks, as well as sweet beverages. The parents receive tailored weekly feedback on their registrations, which are presented as graphical illustrations with bars and text using a “traffic light” approach (green = reached the recommendation, yellow = almost reached the recommendation, red = far from reaching the recommendation). Recommendations for the green traffic lights in the different categories are: at least 60 min of active play and maximum 60 min of screen time per day; 2 pieces of fruit and 2 (child-sized) handfuls of vegetables per day; maximum 7 fixed servings of sweets and snacks per week; and a maximum 300 ml sweet beverages per week.

Finally, the library module contains general facts (presented as questions and answers), recipes and tips on physical activity. The physical activity feature contains tips and inspiration on how to increase activity both indoors and outdoors, and includes both text and videos with different activities and games. The recipe feature has a library of healthy recipes and weekly menus with shopping lists of ingredients that are needed, as well as suggestions on how to simply make common family meals a bit healthier. Finally, this module also includes short videos on healthy eating and parental strategies around food.

### Intervention effectiveness evaluation

The MINISTOP 2.0 trial is a two-arm parallel group randomised controlled trial. In total, 500 children aged 2.5–3 years are being recruited during their routine visit at primary child health care in ≥15–20 health care centres throughout Sweden, covering various socioeconomic areas. Following baseline assessment, parents are randomised (1:1) to the intervention (MINISTOP 2.0 app + standard care) or control group (standard care). Outcome measures will be assessed 6 months post randomisation at a routine visit to child health care.

#### Participants and recruitment

All children who attend the routine visit at 2.5–3 years in primary child health care will be screened for eligibility to participate. To be included, at least one parent must be able to speak and read Swedish, English, Arabic or Somali sufficiently to understand consent information. In addition, the Somali and Arabic versions of the app contain audio-files, to facilitate reading and ensure that parents with limited reading skills can also benefit from the content of the app. The nurses were instructed not to offer trial participation to families where the child is diagnosed with a neurological or endocrine disease or where a parent is suffering from a serious physical or mental illness, which would make the trial too demanding for the family.

**Fig. 2:**
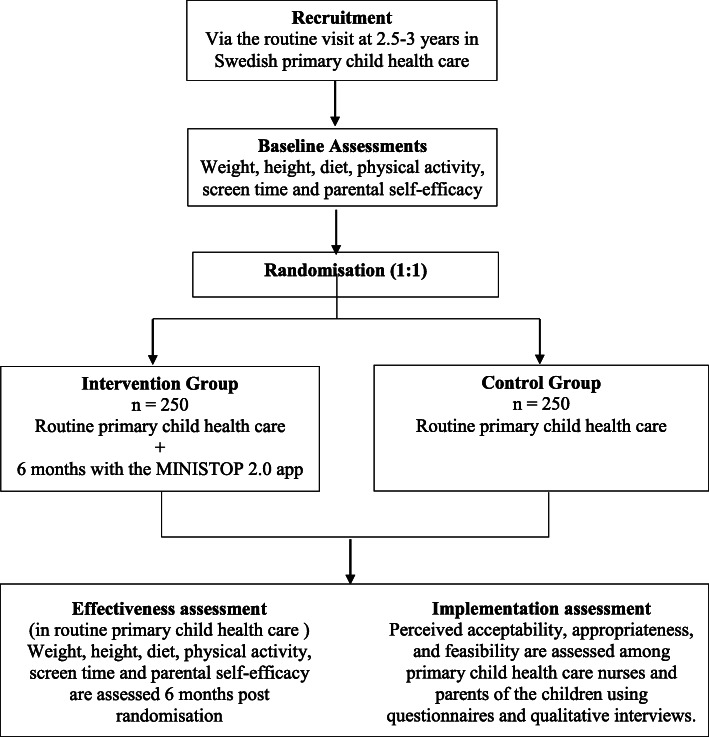
Study design of the MINISTOP 2.0 trial

**Fig. 3:**
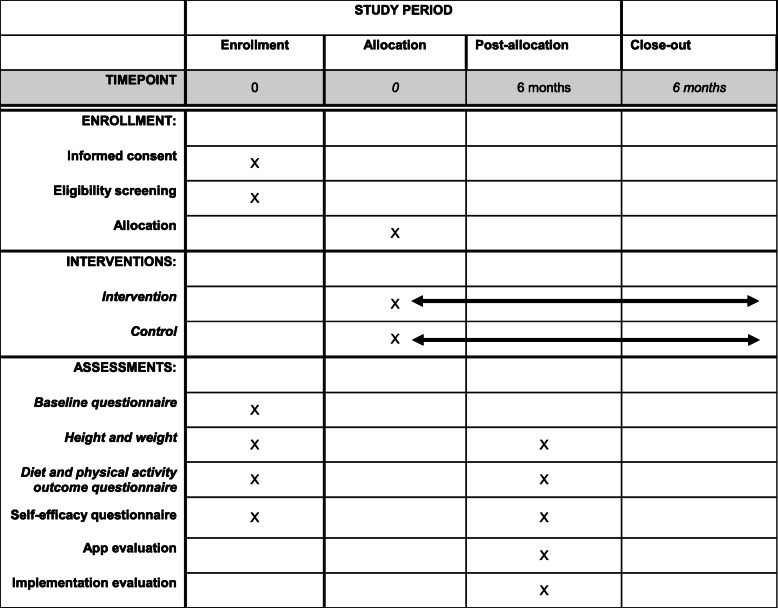
SPIRIT figure presenting participant timeline

Information about the trial is sent to the families’ home addresses together with a routine invitation to visit the primary child health care centre when the child is aged 2.5–3 years. A child health care nurse will ask families about participation in the trial and collect written informed consent from parents. Thereafter, parents complete a questionnaire about the child’s diet, physical activity, screen time, physical fitness and dental health behaviours. The parents also answer questions about their own socioeconomic background as well as questions on parental self-efficacy regarding their own ability for promoting healthy physical activity and dietary behaviours in their child. The nurse also measures the child’s height and weight in a standardised way. Subsequently, participating families are randomised to either an intervention or control group using opaque envelopes. Participants in the intervention group are given access to the MINISTOP 2.0 app for 6 months, in addition to standard care, while those in the control group only receive age-appropriate information about healthy food and physical activity that currently is provided by routine child health care. Six months after randomisation families will attend another routine visit at the primary child health care centre. The nurse again measures the child’s weight and height and the parents complete the same questionnaire about the child’s food, physical activity, screen time, fitness and dental health behaviours. At this visit, the control group will also be offered access to the MINISTOP 2.0 app. Figure 2 outlines the study design and Fig. 3 presents a trial participant timeline.

#### Randomisation and blinding

Following baseline measurements, the children are randomly allocated to either the control group (standard care) or intervention group (standard care + MINISTOP 2.0) in a 1:1 ratio by means of block randomisation generated using R 3.6.1 (by MB). Random block sizes of 2 and 4 were used to ensure that the randomisation could not be subverted. Sealed envelopes are used to allocate children to the respective groups, ensuring allocation concealment. Participants and child care nurses are not blinded to allocation due to the nature of the intervention and recruitment strategy, however the research team will be blinded throughout the trial.

#### Outcome measures

The primary outcomes for the effectiveness of the intervention are diet, physical activity and screen time. BMI and parental self-efficacy are secondary outcomes. Outcomes are assessed at baseline and at 6-months’ follow-up at the primary child health care centres. See Fig. 2 for an overview of all assessments, Fig. 3 for participant timeline and Supplementary material to see all questions used for outcome measures.

##### Health behaviours

The MINISTOP 1.0 app has previously been evaluated with accurate and valid methodology for assessment of body composition, dietary intake and physical activity [28]. In the MINISTOP 2.0 trial, we used a short questionnaire for the outcome measures as all measures are performed by the health care nurses and therefore need to be easily administered. Thus, the questionnaire addresses questions to the parents about their children’s diet, physical activity and screen time. Diet and physical activity are assessed using a modified version of the National Board of Health and Welfare’s survey of health behaviours [40]. Key dietary indicators, i.e. fruit and vegetables, sweet or savoury treats or sugar-sweetened drinks, will be assessed as average number of standardised portions per day during the past month. Physical activity will be assessed as average minutes per day of moderate-to-vigorous physical activity in the past month. Average time spent in front of digital screens (TV, computer, tablets or mobile phones) will be assessed in minutes per day during a typical week and weekend (the past month) based on a questionnaire with established reliability [41]. The questionnaire also contains questions about the child’s dental health behaviours and physical fitness [42]. Finally, the questionnaire addresses questions to the parents about their own age, height and weight, education, country of birth and self-efficacy for promoting healthy physical activity and dietary behaviours in their child [28, 43].

##### BMI

Weight, height and BMI are recorded through standardised measures at the routine visit to primary child health care when children are 2.5–3 years of age. Children are weighed without shoes and with light clothing. BMI is calculated as weight divided by height squared (kg/m^2^) and children are classified into underweight, normal weight, overweight or obese using age- and sex-specific cut-offs established by Cole et al. [44].

#### Data analysis

All analyses will be intention-to-treat, including participants in the groups to which they were randomly allocated. Analyses will first be among complete cases, assuming that any missing data are missing completely at random (MCAR). Attrition and sensitivity analyses will be conducted to assess the plausibility of the MCAR assumption and the effect of potential outliers on the analytical results (Supplementary material). Data input will be checked against source data to ensure that translation from paper questionnaires to digital format did not introduce errors.

Parameters of all models will be estimated using maximum likelihood (MLE) and Bayesian inference [45]. Null hypothesis testing will be conducted at the conventional 0.05 significance threshold. Marginal posterior distributions for coefficients of interest will be examined to calculate the posterior probability of effect (i.e. the proportion of parameters compatible with the MINISTOP 2.0 intervention having a positive effect). The median of the marginal posterior distributions will be used as point estimates of effect. Both null hypothesis testing and Bayesian analysis will create a basis for scientific inference.

Primary outcomes comprising intake of fruit and vegetables, sweet or savoury treats or sugar-sweetened drinks, average minutes per day of moderate-to-vigorous physical activity, and average time spent in front of digital screens (TV, computer, iPad/tablets or mobile phones) will be analysed using normal regression (possibly log transformed if found to be skewed). The secondary outcome of BMI will be analysed using quantile regression (at 10, 50, and 90% quantiles). Furthermore, the secondary outcome of self-efficacy will be analysed using normal regression (possibly log transformed if found to be skewed). All models will include a random intercept accounting for differences among child health care centres, a regressor for group allocation to determine contrasts in the intervention and control groups, adjustment for the baseline value of each respective outcome, and adjustments for age and gender of the child. Effect modification will be analysed by introducing an interaction term between group allocation and baseline variables.

Analyses of measures other than the primary and secondary outcomes will be considered exploratory. We will use regression models with appropriate distributional properties to contrast differences between groups for these outcomes, and findings of consequence will be reported as hypothesis generating.

#### Sample size

With 360 participants (180 per group) completing follow-up, a mere 0.30 standard deviation (SD) difference in outcomes between groups with a power of 80% (α = 0.05) can be detected. This corresponds to differences of 25 g and 0.4 kg/m^2^ in fruit intake and BMI, respectively [23, 28]. We will conduct interaction analyses to examine whether the effect of the intervention differs depending on socioeconomic and migration status. Assuming a maximum dropout rate of ≈ 25–30% [23, 28], 500 children will be recruited. This sample size is considered feasible given our own and other researchers’ previous experiences [23, 28].

### Implementation evaluation

Implementation will be investigated in terms of perceived acceptability, appropriateness, and feasibility concerning the MINISTOP 2.0 app among primary child health care nurses and parents of the children participating in the randomised controlled trial.

#### Participants and data collection

##### Parents

Parents’ satisfaction with and usage of the MINISTOP 2.0 app will be assessed among those in the intervention group, using a modified version of a questionnaire from the MINISTOP 1.0 trial [28]. Proportion of users satisfied with the app and frequency of used features in the app will be assessed. In the questionnaire, all parents are also asked about their interest in participating in individual semi-structured interviews to evaluate perceived acceptability, appropriateness and feasibility of the MINISTOP 2.0 app [46], i.e. three indicators of implementation success [47]. Purposive sampling will be used. Eligible participants will be contacted via telephone whereby a date for an interview will be scheduled. The aim is to recruit approximately 20 participants representing varied age groups, gender, educational levels and country of birth. A semi-structured interview guide will be used including questions that aim to capture parents’ perspectives on using the intervention. Finally, objective data of app usage and participant engagement (such as number of recordings and messages read in the app) will be evaluated to complement the qualitative and self-reported data.

##### Primary child health care nurses

Semi-structured interviews will be conducted with primary child health care nurses to evaluate the following implementation indicators: perceived acceptability, appropriateness and feasibility [46]. All nurses will be invited to take part in interviews via e-mail. Purposive sampling will be used and the aim is to recruit 10–15 participants from various child health care centres to represent different geographical locations and socioeconomic areas. A semi-structured interview guide will be used and questions will aim to explore nurses’ perspectives of using the intervention in routine practice.

#### Data analysis

Interviews with parents and primary child health care nurses will be audio recorded and transcribed verbatim. Data will be analysed using thematic analysis by two independent assessors. The protocol for the interviews has been designed and assessed, in collaboration with experts in qualitative interview methodology (UM, KT).

### Ethics approval

The Swedish Ethical Review Authority (ref no 2019–02747; 2020–01526) approved this research project. Written informed consent is obtained from participating parents.

### Trial status

Recruitment of participants to the MINISTOP 2.0 trial was initiated in November 2019 and the trial is progressing according to plan. This protocol was finalised before the research team had received any data.

## Discussion

The MINISTOP 2.0 trial will evaluate whether an mHealth intervention embedded in the routine services provided by primary child health care can be used to improve diet and physical activity behaviours and prevent overweight and obesity in preschool-aged children. Most previous interventions using digital delivery methods targeting children have been conducted in older children and do not involve parents [48]. The few mHealth intervention trials that have been conducted in children under 3 years of age have had relatively small sample sizes, but have shown promising results [49, 50]. The MINISTOP 2.0 trial is the first study, to the best of our knowledge, to evaluate an intervention delivered by a smartphone app embedded in routine child health care.

Our trial has several strengths including the hybrid type 1 study design, which allows for the testing of the effectiveness of MINISTOP 2.0 and assessment of various implementation aspects. Blending effectiveness and implementation studies provides benefits over carrying out these types of studies independently. This design enables faster knowledge generation and increased usefulness of clinical research if effectiveness is proven [32]. Another strength is the randomised controlled trial design for the assessment of the effectiveness, a planned large sample size, and close collaboration with primary child health care. Participating children are recruited from primary child health care, where almost 99% of all children attend the routine visits [51]) from centres throughout Sweden covering various socioeconomic areas. Thus, the pragmatic blended study design, recruiting in real life conditions with non-restrictive inclusion criteria, increases the generalisability of the trial results. The intervention is grounded in Social Cognitive Theory [35] and uses well-recognised strategies for promoting behaviour change [36]. Considering that ≈25% of all Swedish children have a migrant background [29], another strength of the MINISTOP 2.0 trial is that the app has been translated from Swedish to three other prominent language groups in Sweden (English, Arabic and Somali). We target multiple health-related behaviours in the intervention, including healthy eating, physical activity, screen-time, dental health and sleep. Finally, the trial investigates the perceived acceptability, appropriateness and feasibility among both parents and primary child health care nurses, three aspects that are often considered as key indicators of implementation success [47]. The investigation of the implementation indicators is important for improved understanding of the external validity, i.e. how or the extent to which the results are relevant beyond the trial [52]. Thus, the trial offers the opportunity to generate valuable knowledge for future scale-up and widespread implementation of the app.

There are also some limitations included in the study design. The app has not been translated to all spoken languages in Sweden, and some families who speak minority languages will not be able to participate in the trial. Also, although parents from various socioeconomic backgrounds will be offered participation in the trial, it is possible that there will be an underrepresentation of families with lower education. We will, however, analyse whether there are any differences in intervention effect depending on socioeconomic status or migration background. We use self-reported data for some outcomes, which is common in large studies measuring lifestyle changes [53]. However, the combination of self-reporting and the lack of blinding of participants and nurses, who are responsible for data collection, increases the risk of differential social desirability bias between arms, and research participation effects more generally. This is, however, a consequence of the scale and pragmatic design of the trial, and our findings should be viewed under this limitation. The use of blended effectiveness-implementation designs can be challenging because researchers from clinical and implementation research tend to use different theories, concepts and constructs [32]. However, the clinical and implementation researchers in the MINISTOP 2.0 trial have collaborated from the outset of the trial, thus having developed efficient communication and a shared understanding of both spheres of research.

The MINISTOP 2.0 app has the potential to be implemented in primary child health care nationally, and thereby reach many families who may benefit from it. This is highly relevant considering that obesity is a major public health challenge globally as well as in Sweden. Primary child health care is an important arena for strategies to prevent childhood obesity and also decrease health inequalities [19, 51], as almost all preschool-aged children in Sweden, irrespective of socioeconomic status and migrant background, attend primary child health care [51]. If effective and feasible within the child health care setting, the MINISTOP 2.0 app has the potential to be an evidence-based, low-cost tool to aid primary child health care professionals in their work to promote healthy dietary and activity behaviours, prevent obesity as well as to decrease health inequalities already at preschool age.

## Supplementary Information

**Additional file 1.**

## Data Availability

This article describes a study protocol; thereby data sharing is not applicable as no data are generated or analysed.

## Abbreviations

App: Application
BMI: Body mass index
mHealth: Mobile health
OR: Odds ratio
SD: Standard deviation.
