# Supplementary material for: MINISTOP 2.0: a smartphone app integrated in primary child health care to promote healthy diet and physical activity behaviours and prevent obesity in preschool-aged children: protocol for a hybrid design effectiveness-implementation study
Source: BMC Public Health. 2020 Nov 23;20:1756. doi: 10.1186/s12889-020-09808-w (PMC7687729; doi:10.1186/s12889-020-09808-w)
Supplement: Supplementary file 1 — Additional file 1. [file 12889_2020_9808_MOESM1_ESM.docx]

**Supplementary material**

**MINISTOP 2.0: A smartphone app integrated in primary child health care to promote healthy diet and physical activity behaviours and prevent obesity in preschool-aged children: protocol for a Hybrid Design effectiveness-implementation study**

Hanna Henriksson^1^, Christina Alexandrou^1^, Pontus Henriksson^1^, Maria Henström^2^, Marcus Bendtsen^1^, Kristin Thomas^1^, Ulrika Müssener^1^, Per Nilsen^1^, Marie Löf^1,2^

^1^ Department of Health, Medicine and Caring Sciences, Linköping University, Linköping, Sweden.

^2^ Department of Biosciences and Nutrition, Karolinska Institutet, Stockholm, Sweden.

**Description of supplemental materials:**

1. Questionnaire for parents

#### 2. Attrition and sensitivity analyses

| **Questionnaire for parents (background variables)** | | | |
| --- | --- | --- | --- |
|  | |  | |
| **The child** | |  | |
| The child’s date of birth (dd/mm/yyyy): __________________________________________ | | | |
| The child’s gender: _____________________________________________________________ | | | |
| The child’s country of birth: _______________________________________________________ | | | |
|  | |  | |
| **Guardian 1** | |  | |
| Age: _____________________________ | | Gender: ______________________________ | |
| Current weight (kg):__________________ | | Current height (cm): _______________ | |
| Highest level of education: | |  | |
| ☐ Elementary school | ☐ Upper secondary school | | ☐ College/University |
| Country of birth: ____________________________________________________________ | | | |
| Parent’s country of birth: _____________________________________________________ | | | |
| Language spoken at home: __________________________________________________ | | | |
|  | |  | |
| **Guardian 2** | |  | |
| Age: _____________________________ | | Gender: _______________________________ | |
| Current weight (kg):__________________ | | Current height (cm): ________________ | |
| Highest level of education: | |  | |
| ☐ Elementary school | ☐ Upper secondary school | | ☐ College/University |
|  | |  | |
| Country of birth: ____________________________________________________________ | | | |
| Parent’s country of birth: _____________________________________________________ | | | |
| Language spoken at home: __________________________________________________ | | | |
|  | |  | |
| **Contact information** | |  | |
| Address: _________________________________________________________________  _______________________________________________________________________ | | | |
| Telephone number: __________________________________________________________ | | | |
| Email: __________________________________________________________________ | | | |

| **Questions about your child’s lifestyle habits** | | | | | | | | | | | |
| --- | --- | --- | --- | --- | --- | --- | --- | --- | --- | --- | --- |
| Here are some questions to help us understand your child’s habits. Indicate one answer to each question – what your child normally does. Think back over the past month. | | | | | | | | | | | |
| **Food** | | | | | | | | | | | |
| **1. How many portions of vegetables or root vegetables (fresh, frozen or cooked) does your child eat per day?** *One portion equals about one (child size) handful of vegetables.* | | | | | | | | | | | |
|  3 portions per day or more | | | | | | | | | | | |
|  2.5 portions per day | | | | | | | | | | | |
|  2 portions per day | | | | | | | | | | | |
|  1.5 portions per day | | | | | | | | | | | |
|  1 portion per day | | | | | | | | | | | |
|  3-6 portions per week | | | | | | | | | | | |
|  1-2 portions per week | | | | | | | | | | | |
|  Fewer than one portion per week or none ever | | | | | | | | | | | |
| **2. How many portions of fruits or berries (fresh, frozen, tinned, etc.) does your child eat per day?** *One portion equals 1 normal size fruit or 1 dl of berries or fruit pieces.* | | | | | | | | | | | |
|  3 portions per day or more | | | | | | | | | | | |
|  2.5 portions per day | | | | | | | | | | | |
|  2 portions per day | | | | | | | | | | | |
|  1.5 portions per day | | | | | | | | | | | |
|  1 portion per day | | | | | | | | | | | |
|  3-6 portions per week | | | | | | | | | | | |
|  1-2 portions per week | | | | | | | | | | | |
|  Fewer than one portion per week or none ever | | | | | | | | | | | |
| **3. How often does your child eat fish or shellfish as a main meal?** | | | | | | | | | | | |
|  7 times per week or more | | | | | | | | | | | |
|  6 times per week | | | | | | | | | | | |
|  5 times per week | | | | | | | | | | | |
|  4 times per week | | | | | | | | | | | |
|  3 times per week | | | | | | | | | | | |
|  2 times per week | | | | | | | | | | | |
|  1 time per week | | | | | | | | | | | |
|  Less often than 1 time per week or never | | | | | | | | | | | |
| **4. How often does your child eat or drink one portion of sweet or savoury treats or sweet drink?** *One portion equals around 1 ice cream, 2 biscuits, 1 bun, 0.5 dl of candy, 1 dl of crisps or 3 dl of soft drink/cordial.* | | | | | | | | | | | |
|  3 portions per day or more | | | | | | | | | | | |
|  2.5 portions per day | | | | | | | | | | | |
|  2 portions per day | | | | | | | | | | | |
|  1.5 portions per day | | | | | | | | | | | |
|  1 portion per day | | | | | | | | | | | |
|  3-6 portions per week | | | | | | | | | | | |
|  1-2 portions per week | | | | | | | | | | | |
|  Fewer than one portion per week or none ever | | | | | | | | | |  | |
| **Physical activity** (Please circle the answer you think fits best) | | | | | | | | | | |  |
| **5. On a normal weekday, how much time does your child spend doing physical activity that causes their heart to beat faster and sometimes makes them out of breath, e.g. running, football, jumping, dancing?** | | | | | | | | | | |  |
| 0 min | 15 min | 30 min | 45 min | 1 hour | 1,5 h | 2 h | 2,5 h | 3 h | ≥3,5 hours | |  |
|  |  |  |  |  |  |  |  |  |  | |  |
| **6. On a day off/weekend, how much time does your child spend doing physical activity that causes their heart to beat faster and sometimes makes them out of breath, e.g. running, football, jumping, dancing?** | | | | | | | | | | |  |
| 0 min | 15 min | 30 min | 45 min | 1 hour | 1,5 h | 2 h | 2,5 h | 3 h | ≥3,5 hours | |  |
|  |  |  |  |  |  |  |  |  |  | |  |
| **Screen time** (Please circle the answer you think fits best) | | | | | | | | | | |  |
| **7. How much time does your child spend watching TV or films on a computer, pad or mobile phone?** | | | | | | | | | | |  |
| **a) on a normal weekday?** | | | | | | | | | | |  |
| 0 min | 15 min | 30 min | 45 min | 1 hour | 1,5 h | 2 h | 2,5 h | 3 h | ≥3,5 hours | |  |
|  |  |  |  |  |  |  |  |  |  | |  |
| **b) on a day off/weekend** | | | | | | | | | | |  |
| 0 min | 15 min | 30 min | 45 min | 1 hour | 1,5 h | 2 h | 2,5 h | 3 h | ≥3,5 hours | |  |
|  |  |  |  |  |  |  |  |  |  | |  |
| **8. How much time does your child spend playing games or learning using a computer, pad, mobile phone or video game?** | | | | | | | | | | |  |
| **a) on a normal weekday?** | | | | | | | | | | |  |
| 0 min | 15 min | 30 min | 45 min | 1 hour | 1,5 h | 2 h | 2,5 h | 3 h | ≥3,5 hours | |  |
|  |  |  |  |  |  |  |  |  |  | |  |
| **b) on a day off/weekend** | | | | | | | | | | |  |
| 0 min | 15 min | 30 min | 45 min | 1 hour | 1,5 h | 2 h | 2,5 h | 3 h | ≥3,5 hours | |  |
|  |  |  |  |  |  |  |  |  |  | |  |
|  | | | | | | | | | | |  |
| **Fitness** | | | | | | | | | | |  |
| **9. Try to think about your child’s fitness level (compared to friends of the same age). By fitness level, we mean the child’s ability to participate in long-lasting and pulse-raising activities/games.** | | | | | | | | | | |  |
| **My child’s physical fitness is generally:**   Very bad   Bad   Quite bad   Neither good nor bad   Quite good   Good   Very good | | | | | | | | | | |  |

**Dental health**

**10. How often does an adult brush your child's teeth?**

☐ 2 times per day or more

☐ 1 time per day

☐ A couple of times per week

☐ 1 time per week

☐ A couple of times per month

☐ 1 time per month

☐ Never

**11. Does your child eat or drink something after tooth brushing in the evening (water does not count)?**

 ☐ Yes  ☐ No ☐ Sometimes

**12. Does your child use fluoride toothpaste?**

 ☐ Yes ☐ No ☐ Sometimes

**13. How many times per day does your child eat and/or drink anything (water does not count)?**

☐ 9 times per day or more

☐ 8 times per day

☐ 7 times per day

☐ 6 times per day

☐ 5 times per day

☐ 4 times per day

☐ 3 times per day

☐ 1-2 times per day

**14. How often does your child drink sweet drinks (e.g. soft drinks, juice, cordial, nectar, energy drink, chocolate drink, fruit soup or sweetened drinking yogurt)?**

☐ 4 times per day or more

☐ 3 times per day

☐ 2 times per day

☐ 1 times per day

☐ 5-6 times per week

☐ 3-4 times per week

☐ 1 time per week

☐ Less than one time per week

☐ Never

| **Parents’ belief in their ability to promote good habits in their child**  (Please circle the answer you think fits best) | | | | | | | | | | | | | | | | | | | | | | |
| --- | --- | --- | --- | --- | --- | --- | --- | --- | --- | --- | --- | --- | --- | --- | --- | --- | --- | --- | --- | --- | --- | --- |
|  | | | | | | | | | | | | | | | | | | | | | | |
| **15. How much do you believe in your ability to promote good dietary habits in your child?** | | | | | | | | | | | | | | | | | | | | | | |
| 0 | | 1 | | 2 | | 3 | | 4 | 5 | | | 6 | | 7 | | | 8 | | 9 | | | 10 |
| Not at all |  | | Very little | |  | | A bit | |  |  | Quite a bit | |  | |  | A lot | |  | |  | Very much | |
|  | | | | | | | | | | | | | | | | | | | | | | |
|  | | | | | | | | | | | | | | | | | | | | | | |
| **16. How much do you believe in your ability to promote good activity habits in your child?** | | | | | | | | | | | | | | | | | | | | | | |
| 0 | | 1 | | 2 | | 3 | | 4 | 5 | | | 6 | | 7 | | | 8 | | 9 | | | 10 |
| Not at all |  | | Very little | |  | | A bit | |  |  | Quite a bit | |  | |  | A lot | |  | |  | Very much | |
|  | | | | | | | | | | | | | | | | | | | | | | |
|  | | | | | | | | | | | | | | | | | | | | | | |
| **17. How much do you believe in your ability to limit your child’s screen time (TV, computer, pad, mobile phone, video games)?** | | | | | | | | | | | | | | | | | | | | | | |
| 0 | | 1 | | 2 | | 3 | | 4 | 5 | | | 6 | | 7 | | | 8 | | 9 | | | 10 |
| Not at all |  | | Very little | |  | | A bit | |  |  | Quite a bit | |  | |  | A lot | |  | |  | Very much | |
|  | | | | | | | | | | | | | | | | | | | | | | |
|  | | | | | | | | | | | | | | | | | | | | | | |
| **Please make sure that you have completed the answers to all questions on all pages.** | | | | | | | | | | | | | | | | | | | | | | |
|  | | | | | | | | | | | | | | | | | | | | | | |
| Thanks for your answers! | | | | | | | | | | | | | | | | | | | | | | |

#### Attrition and sensitivity analyses

There are three cases of missingness that may be present in the primary outcomes:

1. Complete missing of primary outcome data for families not visiting the child health care for a second routine visit.
2. Complete missing of primary outcome data for families visiting the child health care for a second visit, but either not willing to complete trial questionnaires or not being asked to do so.
3. Partial missing of primary outcome data for families visiting the child health care for a second visit, but not completing all questions.

Dummy variables will be created to categorize families to one of the three above or a fourth category for completers. If data is available to break up category two into “not willing” and “not asked to“, then this will be done to create a total of five categories. Multinomial regression (with completers as reference) will be used to assess if any of the baseline variables, including the clinic from which families were recruited, are associated with belonging to one of the missingness categories. In addition, associations between missingness of specific outcome variables and baseline variables (in category three) will be analyzed using logistic regression. Models will be regularized (lasso and Bayesian regularization) to identify which baseline variables are influential on missingness. The posterior probabilities of associations, and statistical significance, will be considered in relation to the plausibility of the MCAR (missing data is missing completely at random) assumption and risk of attrition bias will be judged in light of this.

If the evidence against MCAR is judged to be weak, or if there is a plausible explanation that outcome data is random conditional on the baseline variables (ie. missing at random), then we will consider revisiting the primary and secondary analyses with imputation.

Findings from the attrition analyses will be taken into account during the process of scientific inference, and results from outcome analyses judged in light of potential attrition bias. Any evidence of attrition bias will be considered a limitation of the trial.
